# College of General Practitioners

**Published:** 1954

**Authors:** D. M. Cameron

**Affiliations:** Hon. Sec.


### COLLEGE OF GENERAL PRACTITIONERS

### South Western Regional Faculty

In January, 1953, the College was launched, and it quickly became apparent that it was being supported. The Foundation Council from the beginning realized that it must differ from other colleges in that its work must be done in regions, preferably centred on universities. When over a hundred from the South West had become foundation members, it was decided to call a meeting in Bristol. On 28th June, 1953, sixty-four members from the counties of Bristol, Cornwall, Devon, Dorset, Gloucester, Somerset and Wilts met in the Board Room of the Bristol Royal Infirmary. Dr. John Hunt, honorary secretary of the College, addressed the meeting, and after full discussion, it was resolved to form a South Western Regional Faculty. A Board was thereupon elected, consisting of three members from each of the seven counties, and this Board met after the general meeting, and elected as its Chairman Dr. J. H. Grove-White of Cirencester, and as its Vice-Chairman Dr. R. M. S. McConaghey of Dartmouth. It held its second meeting in October, and will meet again in December. Dr. A. H. Gale, Director of Medical Postgraduate Studies at the University of Bristol, was present by invitation at the inaugural meeting of the Faculty, and has been most helpful. Mr. John Dodd has very kindly taken on the duties of Honorary Assistant Secretary.

The aims of the College are not political. They are to watch over the academic interests and education of general practitioners. The Report of the Steering Committee says it " can find no evidence that any existing administrative body in this country is doing now, or will be able to do in the future, what practitioners require ". The main committees of the Foundation Council are concerned with Undergraduate Education, Postgraduate Study and Research. These three subjects will also be the main concern of the Regional Faculties. Any general practitioner who wishes to join the College should write to me.

D. M. CAMERON,

*Hon. Sec.*

### *News from Societies*

